# Gβγ and the C Terminus of SNAP-25 Are Necessary for Long-Term Depression of Transmitter Release

**DOI:** 10.1371/journal.pone.0020500

**Published:** 2011-05-25

**Authors:** Xiao-lei Zhang, Chirag Upreti, Patric K. Stanton

**Affiliations:** 1 Department of Cell Biology and Anatomy, New York Medical College, Valhalla, New York, United States of America; 2 Department of Neurology, New York Medical College, Valhalla, New York, United States of America; The Research Center of Neurobiology-Neurophysiology of Marseille, France

## Abstract

**Background:**

Short-term presynaptic inhibition mediated by G protein-coupled receptors involves a direct interaction between G proteins and the vesicle release machinery. Recent studies implicate the C terminus of the vesicle-associated protein SNAP-25 as a molecular binding target of Gβγ that transiently reduces vesicular release. However, it is not known whether SNAP-25 is a target for molecular modifications expressing *long-term* changes in transmitter release probability.

**Methodology/Principal Findings:**

This study utilized two-photon laser scanning microscopy for real-time imaging of action potential-evoked [Ca^2+^] increases, in single Schaffer collateral presynaptic release sites in *in vitro* hippocampal slices, plus simultaneous recording of Schaffer collateral-evoked synaptic potentials. We used electroporation to infuse small peptides through CA3 cell bodies into presynaptic Schaffer collateral terminals to selectively study the presynaptic effect of scavenging the G-protein Gβγ. We demonstrate here that the C terminus of SNAP-25 is necessary for expression of LTD, but not long-term potentiation (LTP), of synaptic strength. Using type A botulinum toxin (BoNT/A) to enzymatically cleave the 9 amino acid C-terminus of SNAP-25 eliminated the ability of low frequency synaptic stimulation to induce LTD, but *not* LTP, even if release probability was restored to pre-BoNT/A levels by elevating extracellular [Ca^2+^]. Presynaptic electroporation infusion of the 14-amino acid C-terminus of SNAP-25 (Ct-SNAP-25), to scavenge Gβγ, reduced both the transient presynaptic inhibition produced by the group II metabotropic glutamate receptor stimulation, and LTD. Furthermore, presynaptic infusion of mSIRK, a second, structurally distinct Gβγ scavenging peptide, also blocked the induction of LTD. While Gβγ binds directly to and inhibit voltage-dependent Ca^2+^ channels, imaging of presynaptic [Ca^2+^] with Mg-Green revealed that low-frequency stimulation only transiently reduced presynaptic Ca^2+^ influx, an effect not altered by infusion of Ct-SNAP-25.

**Conclusions/Significance:**

The C-terminus of SNAP-25, which links synaptotagmin I to the SNARE complex, is a binding target for Gβγ necessary for both transient transmitter-mediated presynaptic inhibition, and the induction of presynaptic LTD.

## Introduction

Activity-dependent, long-term changes in synaptic strength, such as LTP and LTD, are believed to be important for information storage, neural network development, fine-tuning of synaptic connections, and learning and memory [1], [2], [3], [4]. A wealth of studies have supplied evidence of both presynaptic and postsynaptic long-term alterations associated with LTP and LTD [5], [6], [7]. Postsynaptic alterations include changes in AMPA receptor-gated channel conductance [8], [9] insertion/removal of AMPA receptors [10], [11] and changes in dendritic spine shape [12], [13]. Evidence of presynaptic changes has been derived largely from quantal analysis studies of pairs of monosynaptically-connected neurons [14], [15], [16] vesicular antibody uptake [17] and postsynaptic drug infusion ([18], [19], [20]. More recently, we and others have used two-photon imaging of the vesicle-specific styryl dye FM1-43 to demonstrate directly that LTP and LTD can both be associated with long-term changes in transmitter release [21], [22], [23], [24] but that sub-maximal LTP can also be induced without any change in presynaptic release probability [23], supporting the notion that postsynaptic and presynaptic long-term changes can occur independently and be mediated by different cellular biochemical cascades.

Previously, we have shown that activation of G protein-coupled receptors (GPCRs) for glutamate and adenosine is necessary for the full induction of LTD at Schaffer collateral-CA1 synapses [25], and that pairing the generation of cyclic GMP with either activation of these GPCRs [25] or an inhibitor of cyclic AMP-dependent protein kinase [26], is *sufficient* to elicit LTD. Furthermore, LTD can be elicited in slices from mice over-expressing a constitutively-active Giα by simply elevating [cyclic GMP], indicating that one key role of GPCRs in promoting the induction of LTD is the inhibition of adenylate cyclase [27].

However, activating GPCRs negatively coupled to adenylate cyclase leads to release of *both* Giα and Gβγ moieties, in a 1:1 stoichiometric ratio [28]. Alford and colleagues have shown at synapses in the lamprey [29], [30], and superior cervical ganglion [31], that Gβγ released by GPCRs (noradrenergic and serotonergic receptors) mediate presynaptic inhibition by binding to the c-terminus of the SNARE protein SNAP-25 [29].

In the present study, we tested the hypothesis that the Gβγ released along with Giα by GPCR activation may also be a necessary step in the transition from transient presynaptic inhibition of release to presynaptic LTD. We found that cleavage of the 9 amino acid C-terminus of SNAP-25 with botulinum toxin A, or presynaptic infusion of either the 15 amino acid C-terminus of SNAP-25 or the Gβγ binding peptide mSIRK, each significantly reduced the magnitude of LTD induced at Schaffer collateral-CA1 synapses, consistent with Gβγ binding to the C-terminus region of SNAP-25 being an important step in the expression of presynaptic LTD of vesicular transmitter release.

## Materials and Methods

### Ethics Statement

All experiments were performed under an approved protocol from the Animal care and Use Committee of New York Medical College, in compliance with National Institutes of Health Guidelines for Animal Use.

### Drugs

All external and patch pipette solutions were made with deionized distilled water (resistance >18 MΩ cm^−2^; Milli-Q system). The chemicals for making extra- and intracellular solutions were purchased from Sigma-Aldrich. Neurotransmitter receptor antagonists were purchased from Tocris Cookson Ltd.; Alexa Fluor 594, Magnesium Green and FM4-64 were purchased from Molecular Probes, and mSIRK was purchased from EMD Chemicals Group. BoNT/A was purchased from Sigma-Aldrich and stored at -20C until the day of the experiment, when it was pre-incubated in ACSF containing 5 mM dithiothreitol (DTT) for 30 minutes prior to application, to reduce and thereby activate the toxin.

### Slice Preparation and Extracellular Recordings

Twelve-17 day old Sprague-Dawley rats (Taconic) were decapitated under deep isoflurane anesthesia, the brains quickly removed, hemisected, and a tissue block containing the hippocampus prepared. The block was glued to a stage immersed in ice-cold oxygenated ACSF (2–4°C), and 400 µm thick transverse hippocampal slices cut with a vibratome (DSK model DTK-1000). Slices were placed in an interface holding chamber containing ACSF at room temperature for at least one hr, and then transferred to an interface chamber for recording at 32°C. Slices were perfused with artificial cerebrospinal fluid (4 ml/min; ACSF mM: NaCl 126; KCl 3; NaH_2_PO_4_ 1.25; MgCl 1.3; CaCl_2_ 2.5; NaHCO_3_ 26; glucose 10) saturated with 95%O_2_/5%CO_2_, and all drugs were bath-applied. Low resistance recording electrodes were pulled with a Flaming/Brown Micropipette puller (Model P-97, Sutter Instrument) using thin-walled borosilicate glass (1–2 MΩ after filled with ACSF) and inserted into the stratum radiatum of field CA1 region, to record field excitatory post-synaptic potentials (fEPSPs). A bipolar stainless steel stimulating electrode (FHC Co.) was placed in Schaffer collateral-commissural fibers in the CA3 region, and current pulses were applied with stimulus intensity adjusted to evoke approximately 50% of maximal fEPSPs once each 30 s (50 to 100 pA; 100 µs duration). Electrical stimulation from an ISO-Flex isolator was controlled by a Master eight-pulse generator (AMPI, Jerusalem, Israel) and triggered by a Multiclamp 700B (Molecular Devices, Sunnyvale, CA). Signals were digitized with a Digidata 1322 and recorded using a Multiclamp 700B amplifier. fEPSP slope was measured by linear interpolation from 20–80% of maximum negative deflection, and slopes confirmed to be stable to within 10% for at least 15 min before commencing an experiment. Data were analyzed using Clampfit (Version 9; Axon Instrument) on an IBM-compatible personal computer. Evoked fEPSPs (50% of maximum amplitude, 2–4 mV) were recorded in the apical dendritic field in *stratum radiatum* for a stable baseline period of at least 30 min. The stimulus paradigm for induction of homosynaptic LTD by low-frequency stimulation (LFS) was as used by Dudek and Bear (1992). SLTD Stimulation consists of 1200 constant current square pulse stimuli (150 µsec duration each), given at a frequency of 2 Hz, for a LFS duration of 10 min.

### Electroporation of Peptides into Presynaptic CA3 Pyramidal Neurons

Patch pipettes (3–4 MΩ filled with ACSF) containing 1 mM Alexa Fluor 594 plus 1 mM Ct-SNAP-25 peptide were inserted into the pyramidal cell layer in the CA3 region of *in vitro* hippocampal slices, and pyramidal neurons electroporated using positive voltage pulse (30 ms, 30 volts). 10∼15 pulses were delivered at a frequency of 0.5 Hz to each electroporation site, starting at the end of the CA3 region closest to the dentate hilus, with the pipette inserted 50 µm below the slice surface. The pipette was moved two more times in the vertical axis in 75 µm steps with each step receiving a series of electroporation pulses (10∼15 pulses @ 0.5 Hz). The pipette was then moved 20 µm horizontally to another site in CA3 *stratum pyramidale* and the electroporation depth series repeated, continuing until the CA3-CA2 border is reached (9–12 sites). After the electroporation protocol was completed, slices were moved to a holding chamber for at least 45 minutes before transfer to the recording chamber to start the experiment.

### Presynaptic Infusion of the Cell-Permeant Gβγ Binding Peptide mSIRK into CA3 Pyramidal Neurons

Focal injections of mSIRK were performed with a Picosprizer II (General Velve Corp. NJ), from 0.8–1 MΩ resistance injection pipettes filled with ACSF containing 100 µM mSIRK in 1% DMSO, inserted into *stratum pyramidale* of field CA3 to a depth of 100 µm. Each injection consisted of two 500 msec pulses 10 sec apart at a pressure of 3 psi, and 8–10 injection points were used at 50 µm intervals to cover the entire CA3 region as shown in the inset of figure 6D. The direction of perfusion of slices with ACSF was from distal end of CA1 towards CA3, so that an mSIRK peptide that remained extracellular would wash away from field CA1.

### Presynaptic [Ca^2+^] imaging

Fluorescence was visualized using a customized two-photon laser-scanning Olympus BX61WI microscope with a 60x/0.90W water immersion infrared objective lens and an Olympus multispectral confocal laser scan unit. The light source was a Mai-Tai™ laser (Solid-State Laser Co., Mountain View, CA), tuned to 820 nm for exciting Magnesium Green and Alexa Fluor 594, and 910 nm for exciting FM4-64. Epifluorescence was detected with photomultiplier tubes of the confocal laser scan head with pinhole maximally opened and emission spectral window optimized for signal over background. In the transfluorescent pathway, a 565 nm dichroic mirror was used to separate green and red fluorescence to eliminate transmitted or reflected excitation light (Chroma Technology, Rockingham, VT). Depending on the natures of the fluorescent dyes, HQ525/50 and HQ610/50 or HQ710/50 filters were placed in the “green” and “red” pathways, respectively. Image acquisition was controlled by Fluoview FV300 software (Olympus America, Melville, NY). Alexa Fluor 594 was loaded into CA3 pyramidal neurons with electroporation to show the feasibility of loading Ct-SNAP-25 into CA3 pyramidal neurons with the same method. Using a well-established technique [66], we filled Schaffer collateral presynaptic fibres with Magnesium Green AM. Briefly, an ejection electrode (tip diameter, 5–10 µm) containing Magnesium Green AM (1 mM Magnesium Green AM, 10% DMSO, 1% pluronic acid in ACSF) was lowered into the Schaffer collateral pathway between the stimulating electrode and the presynaptic terminal field to be observed, air pressure pulses (6–9 psi, 100–200 ms) controlled by a Picospritzer (General Valve Corp. USA) were applied to the electrode until a small bright spot (≈10 mm in diameter) was observed. Then the slice was maintained with a 3 ml/min flow of oxygenated ACSF for ∼30 minutes to allow the dye to sufficiently diffuse into presynaptic boutons. To verify that magnesium green selectively loaded into presynaptic terminals, FM4-64 was loaded with high K^+^ [21] at the end of each experiment. To measure Ca^2+^ dynamics, the fluorescence was collected by scanning at 200 Hz in a surface-scanning mode (XYT). Baseline fluorescence (F_0_) was the average of four images during control, *Δ*F/F was calculated as (*Δ*F/F)_(*t*)_ = (F_(*t*)_-F_0_)/F_0_.

### Data Analysis

Recording signals were filtered through an eight-pole Bessel low-pass filter with a 1 kHz cutoff frequency and sampled by Clampex (V. 9) with an interval of 100 µs. After fEPSP slopes were calculated with Clampfit (V.9), the data were further processed with Origin 6.1 (Microcal Software, MA) and presented with CorelDraw 10 (Corel, Ottawa, Ontario, Canada).

### Statistical analyses

All data were analyzed by one-way analysis of variance (ANOVA), or Student's t-test using SPSS software (SPSS Inc., Chicago, IL). Significance level was preset to *P *<0.05. Data are presented as mean ± SEM across experiments.

## Results

### Botulinum Toxin type A occludes the induction of stimulus evoked LTD, but not LTP, at Schaffer collateral-CA1 synapses

**Figure 1:**
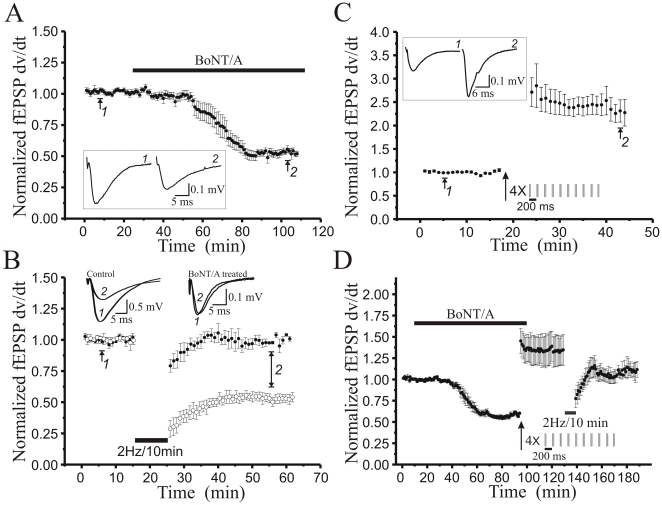
Botulinum Toxin type A pretreatment markedly reduces induction of LTD, but not LTP, at Schaffer collateral-CA1 synapses. **A**: Time course of reduction in field excitatory postsynaptic potential (fEPSP) slopes recorded in CA1 *stratum radiatum* and induced by bath application of BotoxA (200 ng/ml, 90 min; grey bar), plotting normalized fEPSP slopes (*n* = 9 slices). **B**: Time course of the effect of low frequency Schaffer collateral stimulation (2 Hz/10 min, solid bar) on fEPSP slopes in field CA1 of slices (*n* = 6) pretreated for 90 min with BotoxA (200 ng/ml), which eliminated LTD (*P*<0.01; Student’s t-test compared to untreated slice LTD). **C**: Time course of LTP elicited in field CA1 by four trains of theta burst Schaffer collateral stimulation (arrow; each train 10 bursts of 4 stimuli at 100 Hz frequency, 200 ms interburst interval) in slices (*n* = 6) pretreated for 90 min with BotoxA (200 ng/ml). **D**: Time course of the magnitude of depotentiation, where BotoxA (200 ng/ml, grey bar) was bath applied for 90 min prior to induction of LTP by high-frequency theta burst stimulation (arrow; 4 trains of 10 4×100 Hz bursts, 200 ms interburst interval). After LTP was established for 30 min, low frequency Schaffer collateral stimulation (2 Hz/10 min, solid bar) elicited significant depotentation reversal of LTP (*n* = 6). Each point mean ± SEM of *n* slices. Representative fEPSP waveforms insets recorded at times indicated by numbers on traces and time course.

Botulinum toxin type A (BoNT/A) partially suppresses vesicular transmitter release [29], [32] by cleaving 9 amino acids from the C-terminus of SNAP-25 [33], [34], a t-SNARE protein that modulates vesicular release at excitatory synapses. To determine whether the C-terminus of SNAP-25 might contain a regulatory site necessary for long-term plasticity of transmitter release, we applied BoNT/A (200 ng/ml) to hippocampal slices, which produced a slow reduction in evoked fEPSP slope and amplitude which took ∼1 hr to plateau at ∼50% of baseline fEPSP values (Fig. 1A). To test the importance of the C-terminus of SNAP25 to LTD, we preincubated hippocampal slices for 90 minutes with BoNT/A (200 ng/ml) and then applied a 2 Hz/10 minute low frequency Schaffer collateral stimulus (LFS) train previously shown to induce robust LTD [35], [36]. Pretreatment with BoNT/A completely inhibited the induction of stimulus evoked LTD (Fig. 1B; *P*<0.05, Student's t-test compared to control LTD 30 min post-LFS), while not altering transient LFS-evoked depression in these slices. The occlusion of LTD by BoNT/A pretreatment indicates that the C-terminus of SNAP-25 containing residues 198–206 is critical for the induction of stimulus evoked LTD in acute hippocampal slices.

To further probe the contribution of this region of SNAP-25 to long-term synaptic plasticity, we investigated the effect of BoNT/A on the induction of LTP. Surprisingly we noted that pretreatment with BoNT/A did not prevent LTP induction by a theta burst protocol (Fig. 1C; *P*>0.20, Student's t-test compared to control LTP, data not shown), suggesting a selective role for the BoNT/A sensitive region in the C-terminus of SNAP-25 in the induction of LTD.

Next, we investigated the effect of BoNT/A on depotentiation and, as shown in figures 1A and 1C, the presence of BoNT/A led to a depression of fEPSP slopes, that showed robust potentiation following theta burst stimulation (Fig. 1D). Fifteen minutes after the induction of LTP, we elicited depotentiation using 2 Hz/10 minute LFS, which led to a significant and persistent decrease in fEPSP slope (∼30%; Fig. 1D; *P*<0.05, paired t-test compared to pre-LFS LTP), reinforcing the conclusion that the molecular mechanisms for the induction of depotentiation are different from those of long-term synaptic depression and do not require the BoNT/A sensitive C-terminus region of SNAP-25.

### Prior induction of LTD occludes the ability of Botulinum Toxin type A to reduce release probability (Pr), and LTP restores that ability

**Figure 2:**
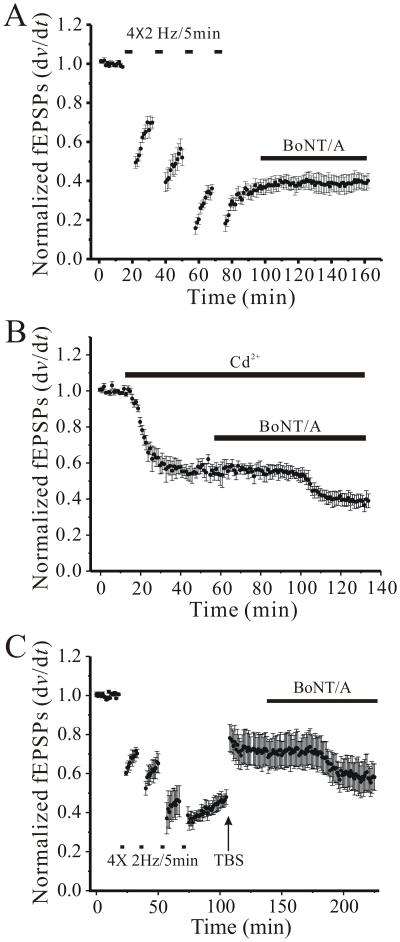
Saturating LTD occludes BoNT/A actions, while reversing LTD with LTP restores BoNT/A efficacy. **A**: Time course of the lack of effect of BoNT/A (200 ng/ml; solid bar) on fEPSP slopes (each point mean ± SEM of 8 slices) after saturation of LTD by four 2 Hz/5 min low frequency Schaffer collateral stimulus trains spaced 15 minutes apart. **B**: Time course of the depression of fEPSPs elicited by BoNT/A (200 ng/ml) after bath application of Cd^2+^ (5 µM) to reduce Pr by ∼50% in 8 slices. **C**: Time course in 8 slices of depression of fEPSPs by BoNT/A (200 ng/ml, bar) elicited after saturating LTD (4×2 Hz/5 min trains), then reversing it with induction of LTP by high-frequency theta-burst stimulation (TBS; 4x trains of 10 bursts of 5pulse/100 Hz each, 200 ms interburst interval).

To further test the hypothesis that LTD is mediated, at least in part, by an action requiring the C-terminus of SNAP-25, we next applied four Schaffer collateral LFS trains (2 Hz/5 min each) to saturate LTD, followed by bath application of BoNT/A. As shown in figure 2A, saturation of LTD completely occluded the action of BoNT/A on synaptic transmission, consistent with an involved of the C-terminus of SNAP-25 in both phenomena. In contrast, when we bath applied a low concentration of Cd^2+^ (5 µM) to reduce fEPSP amplitudes by approximately 50%, BoNT/A was still able to further depress synaptic transmission (Fig. 2B), confirming that simply reducing Pr is not sufficient to explain the occlusion of BoNT/A actions by LTD. Interestingly, when we applied multiple LFS trains to occlude LTD, followed by a high frequency tetanus to elicit LTP to partially reverse LTD, BoNT/A recovered its ability to depress transmission, suggesting that LTP acts directly to reverse SNAP-25 C-terminus dependent mechanisms that occur during LTD (Fig. 2C). While these experiments do not suggest that all of LTD or LTP are expressed presynaptically, they do indicate that a presynaptic component of both LTD and LTP require the C-terminus of SNAP-25, while additional forms of LTP do not.

### BoNT/A reduction in LTD is not due to reduced transmitter Pr

**Figure 3:**
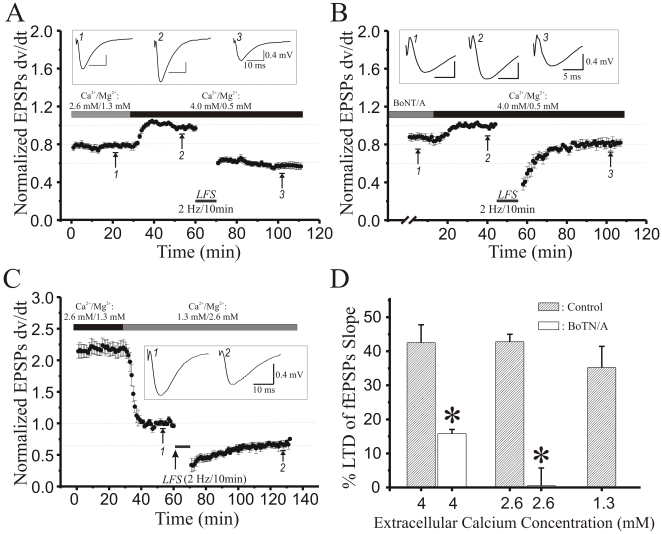
Altering extracellular [Ca^2**+**^]o shows that BotoxA reduction in LTD is not due to reduced transmitter release probability. **A**: Time course of the increase in normalized fEPSP slope (each point mean ± SEM of 8 slices) elicited by raising extracellular [Ca^2+^]o from 2.6 mM (light gray bar) to 4 mM (dark gray bar). After responses had plateaued in high [Ca^2+^]o, a low-frequency stimulus train (2 Hz/10 min, black bar) elicited marked LTD. **B**: Time course of the effect on fEPSP slope of raising extracellular [Ca^2+^]o to 4 mM (dark gray bar), and of LTD elicited by LFS (2 Hz/10 min, black bar), in slices (*n* = 4) pretreated for 90 min with BotoxA (200 ng/ml). Significantly less LTD was evoked (*P*<0.05, Student’s t-test compared to untreated control slices in A). **C**: Time course of the effect of lowering [Ca^2+^]o from 2.6 mM (dark gray bar) to 1.3 mM (light gray bar), and of LTD elicited by LFS (2 Hz/10 min, black bar, *n* = 6). **D**: Mean ± SEM % LTD of Schaffer collateral-evoked fEPSP in *stratum radiatum* of field CA1 in slices treated with 4, 2.6 or 1.3 mM [Ca^2+^]o (hatched bars), compared to slices pre-treated with BoNT/A in 4 or 2.6 mM [Ca^2+^]o (open bars). BoNT/A-treated slices exhibited significantly less LTD than slices in either 4 mM [Ca^2+^]o (*, *P*<0.01; Student’s t-test) or 2.6 mM [Ca^2+^]o (*, *P*<0.05, Student’s t-test).

Previous research has shown that binding of Gβγ proteins to the C-terminus of SNAP25 underlies GPCR-mediated presynaptic inhibition of transmitter release [29]. However, it is also known that Gβγ can bind to voltage-gated calcium channels and directly reduce their conductance, thereby suppressing neurotransmission [37], [38]. To investigate if BoNT/A mediated depression is caused by reduced calcium infux or via an alternative mechanism that involves binding of Gβγ proteins elsewhere, we increased extracellular [Ca^2+^] from 2.6 mM to 4.0 mM, while reducing extracellular [Mg^2+^] to keep total divalent cation concentration equal. A greater electrochemical drive for Ca^2+^ should increase Ca^2+^ influx through VGCCs, restoring release probability (Pr) to pre BoNT/A treatment levels. In control slices, elevating [Ca^2+^]o to 4 mM (inset trace 2, Fig. 3A) produced a significant increase in fEPSP amplitude, consistent with an increase in Pr. Once this baseline stabilized, a low frequency stimulus train (LFS, 2 Hz/10minutes) was sufficient to induce robust LTD of synaptic strength (∼40%, inset trace 3, Fig. 3A). When we pretreated hippocampal slices for 90 minutes with 200 ng/ml BoNT/A in nACSF (2.6 mM [Ca^2+^]), fEPSP amplitude dropped by ∼20% (Fig. 3B), a decrease that was restored by raising extracellular [Ca^2+^]o to 4 mM (dark gray bar). A low frequency Schaffer collateral stimulus train applied in 4 mM [Ca^2+^]o to BoNT/A pretreated slices induced half the magnitude of LTD (Fig. 3B,D) compared to untreated controls (Fig. 3A,D, *P*<0.05, Student’s t-test). This suggests that, even under conditions where Pr was restored to pre-BoNT/A levels, BoNT/A still impaired the expression of LTD, consistent with the C-terminus of SNAP-25 being a necessary target in the induction of LTD.

Next, we tested whether LTD could be induced when Pr was reduced by lowering [Ca^2+^]o to 1.3 mM, which reduced Pr by an amount similar to BoNT/A treatment (mean-variance analysis as in 39; BoNT/A Pr  = 0.12±0.03, 1.3 mM [Ca^2+^]o Pr  = 0.17±0.05). As shown in figure 3C, a 2 Hz/10 min LFS elicited LTD whose magnitude was not significantly different from control LTD (Fig. 3D; *P*>0.20, Student’s t-test), confirming that BoNT/A impairs LTD by some mechanism that requires the C-terminus region of SNAP-25, beyond simply reducing release probability.

### BoNT/A reduces release probability by a mechanism distinct from elevating [cyclic GMP]

Induction of homosynaptic LTD in the CA1 region of hippocampus can be achieved using prolonged periods (10-15 min) of low frequency (1–2 Hz) stimulation (LFS) of Schaffer collateral axons [35], [36]. This form of LTD is blocked by NMDA receptor antagonists [40], and has both postsynaptic [41] and presynaptic components of expression [21], [36]. Previous work has shown that calmodulin-mediated activation of nitric oxide (NO) synthase in the postsynaptic compartment [42], [43] leads to the production of NO that behaves as a retrograde messenger by diffusing out of the postsynaptic compartment and activating a soluble guanylyl cyclase that generates cyclic GMP in the presynaptic terminal [43], leading to decreased transmitter release. Further evidence in support of cGMP-mediated presynaptic depression came from studies showing that pairing an increase in [cGMP] with inhibition of PKA [21], [26], [44] produces LTD of vesicular release of FM1-43 from presynaptic terminals and, in particular, from the rapidly-reycling vesicle pool [21], [22]. Though these data provide strong evidence for the involvement of a ‘NO-cGMP-PKG’ pathway in the induction of a presynaptic component of LTD, the downstream targets of this mechanism still remain elusive.

**Figure 4:**
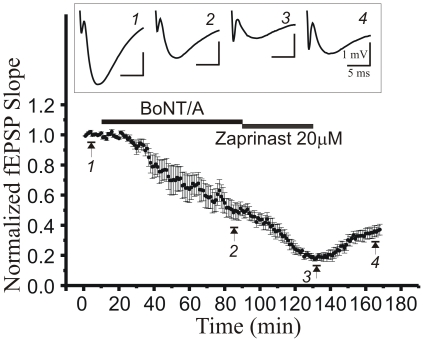
BotoxA reduces release probability by a mechanism distinct from that of elevating [cyclic GMP]. Time course of the additive effects of bath application of BotoxA (200 ng/ml, light gray bar), followed by the type V phosphodiesterase inhibitor zaprinast (20 µM, dark gray bar), on Schaffer collateral-evoked fEPSP slopes in field CA1. Each point is mean ± SEM fEPSP slope from 5 slices. Insets show representative fEPSPs recorded in CA1 *stratum radiatum* at times indicated by corresponding numbers on traces and time course.

To investigate whether the 9 amino acid C-terminus of SNAP-25 is required for cGMP- mediated depression [26], we bath-applied zaprinast (a cGMP-specific phosphodiesterase type V (PDE5) inhibitor), which selectively elevates intracellular [cGMP]. As shown in figure 4, application of zaprinast following BoNT/A treatment caused an additional decrease of fEPSP slope by ∼50%, which recovered after washout of zaprinast, consistent with the reversible nature of this depression triggered due to a transient elevation of [cGMP] [26]. These results suggest that the C-terminus region of SNAP-25 and cGMP-mediated weakening of synaptic strength have different mechanisms of expression.

### Presynaptic infusion of the 14 amino acid C-terminus of SNAP-25 blocks mGluR-dependent presynaptic depression and stimulus evoked LTD

Earlier work from our lab has implicated the Gαi2 mediated inhibition of adenyl cyclase as playing an important role in presynaptic LTD [27]. In the above study, a transient increase in cGMP levels helped unmask LTD in mice expressing a constitutively active Gαi2 [27]. Our data in this study (Fig. 4) suggest that there are different mechanisms by which cGMP mediated depression and stimulus-evoked LTD are expressed. Activation of G-protein coupled receptors leads to a simultaneous release of Gβγ subunits, that dissociate from Gα and are capable of functional interactions with neighboring proteins. We hypothesized that, in addition to Gα-mediated inhibition of adenylyl cyclase, Gβγ subunits might target mechanisms that affect release of neurotransmitter and contribute to LTD. Gβγ proteins are know to bind the C-terminus of SNAP-25 and inhibit transmitter release by a mechanism that involves Gβγ competing with calcium bound synaptotagmin for a binding site on the C-terminus of SNAP-25 [29], [45], thus interfering with vesicle release. We reasoned that binding of Gβγ to SNAP-25 residues 198–206 maybe also be a necessary step for the presynaptic component of LTD of transmitter release.

**Figure 5:**
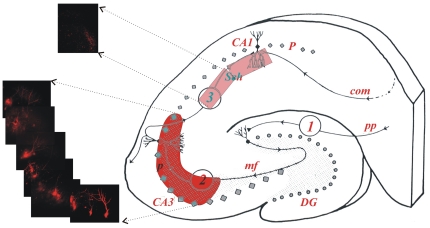
Selective filling of presynaptic CA3 pyramidal neurons using Electroporation. Schematic of a hippocampal slice illustrating in red the region where peptides were infused by electroporation into somata of multiple CA3 pyramidal neurons, and in pink the region where Schaffer collateral presynaptic terminals were imaged to confirm successful fill. Blunt patch electrodes were filled with 1 mM AlexaFluoro-594, and trains of 10 square current pulses (30 V/200 ms, 0.5 Hz) were applied at 50, 100, and 150 µm depth, after which the electrode was removed from the slice, shift laterally 20 µM, and the depth electroporations were repeated. One hour after electroporation, two-photon laser scanning microscopy (63x objective) was used to acquire the attached images of CA3 pyramidal neuron cell bodies (lower left) and Schaffer collateral terminals (upper left) to confirm successful presynaptic infusion.

To directly test the potential role of Gβγ in presynaptic LTD, we used the multiple electroporation method of Haas et al. [46] to selectively load many presynaptic CA3 pyramidal neurons (Fig. 5) with the 14 amino acid cleaved product of the C-terminus of SNAP-25 (**rat Ct-SNAP-25, residues 193–206, DEANQRATKMLGSG**) previously show to be a potent scavenger of free Gβγ [29]. Alexa-Fluoro-594 (1 mM) was included in the patch pipette along with the Ct-SNAP-25 peptide (1 mM) and injected into multiple regions (depicted in red, Fig. 5) of field CA3 in the hippocampal slice using multiple trains of square current pulses [46]. Successful presynaptic infusion of the peptide was verified using two photon imaging of field CA1 (Fig. 5, pink region), one hour after electroporation. During the two hours allowed for presynaptic infusion prior to attempting to induce LTD, the time course of evoked fEPSPs confirmed that Ct-SNAP-25 infusion into CA3 pyramidal neuron terminals had no long-term effects alone on evoked synaptic potentials (data not shown).

**Figure 6:**
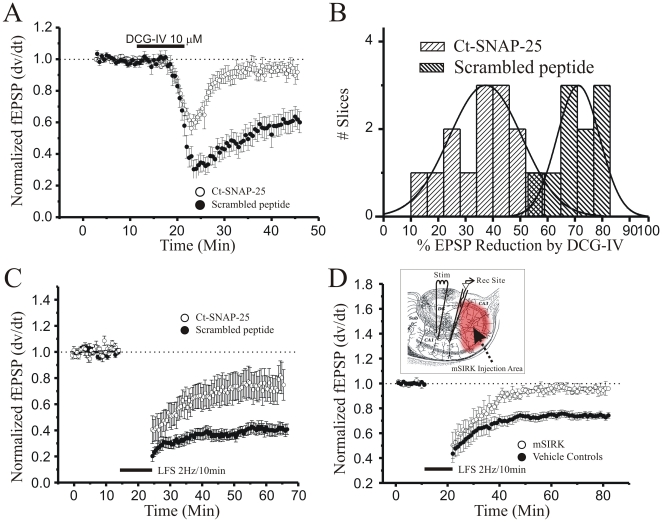
Presynaptic infusion of the C-terminus of SNAP-25 (Ct-SNAP-25) blocks mGluR-dependent LTD and impairs stimulus-evoked LTD, and presynaptic infusion of the Gβγ binding peptide mSIRK also blocks stimulus-evoked LTD. **A**: Effect of electroporation of Ct-SNAP-25 into CA3 pyramidal neurons on DCG-IV induced presynaptic depression of synaptic transmission at Schaffer collateral-CA1 synapses. Plot of the changes in normalized Schaffer collateral-evoked fEPSP slopes (dV/dt) produced by bath application of the group II mGluR agonist DCG-IV (10 µM, solid bar) in slices 2 hr after presynaptic infusion of Ct-SNAP-25 (open circles, *n* = 15) versus infusion of scrambled peptide (filled circles, *n* = 10). Each point mean ± SEM fEPSP slope. **B**: Histogram showing the raw distribution of % fEPSP slope reductions evoked by DCG-IV in Ct-SNAP-25 treated (light hatched bars, bin  = 6%) versus scrambled peptide controls (dark hatched bars). **C**: Effect of presynaptic electroporation infusion of Ct-SNAP-25 (open circles, *n* = 11) versus scrambled peptide (filled circles, *n* = 8) on LTD of Schaffer collateral-evoked fEPSPs elicited by low frequency Schaffer collateral stimulation (LTD; solid bar, 2 Hz/10 min). Each point mean ± SEM fEPSP slope. **D**: Effect of presynaptic infusion of the membrane-permeable Gβγ binding peptide mSIRK (100 µM, open circles, *n* = 6) versus vehicle controls (filled circles, *n* = 8, 1% DMSO in ACSF) on LTD of Schaffer collateral-evoked fEPSPs elicited by low frequency Schaffer collateral stimulation (LTD; solid bar, 2 Hz/10 min).

To characterize the functional role of Gβγ release in GPCR-mediated presynaptic actions, we evoked group II mGluR-dependent LTD by bath application of DCG-IV, a specific agonist for group II metabotropic glutamate receptors, two hours after the presynaptic infusion of either Ct-SNAP-25 peptide or a scrambled control peptide (GQAMGKSNATDREL). As shown in figure 6A, bath application of DCG-IV in the presence of the scrambled peptide lead to a significant depression of synaptic strength (n = 15; −63% ±6% of pre-DCG-IV baseline fEPSPs; *P*<0.05, paired t-test) which persisted for at least 30 minutes after drug washout. Interestingly, DCG-IV mediated depression after electroporation with Ct-SNAP-25 was significantly reduced in magnitude (n = 10; −26% ±5% of pre-DCG-IV baseline fEPSPs; *P*<0.05, Student’s t-test compared to DCG-IV alone) and readily reversed to baseline levels within 10 minutes of drug washout. Cumulative distribution histograms (Fig. 6B) plotting percent fEPSP reduction caused by presynaptic electroporation of Ct-SNAP-25 confirmed the role of this peptide in the inhibition of fEPSP slopes by group II mGluRs (shown here as a left shift in distribution of % fEPSP reduction by DCG-IV). These data suggest that scavenging of Gβγ at Schaffer collateral presynaptic release sites by Ct-SNAP-25 can reverse group II metabotropic glutamate receptors mediated reduction in transmitter release. Additionally, they also show that small peptides and synthetic compounds can be selectively infused into the Schaffer-collateral presynaptic terminals by electroporation at sufficient concentrations to have notable effects.

Next, we investigated the effects of Ct-SNAP-25 infused into presynaptic release sites of CA3 pyramidal neurons on the induction of stimulus-evoked LTD at Schaffer collateral-CA1 synapses. A low frequency 2 Hz/10 minute stimulus train was applied to Schaffer collaterals to elicit LTD 1–2 hours after electroporation of Ct-SNAP-25 or scrambled peptide. As shown in figure 6C, electroporating the scrambled peptide into Schaffer collateral terminals allowed the induction of robust LTD (n = 8; 41±4% fEPSP slope of pre-stimulus baseline 40 min post-LFS), that was not significantly different from control LTD (*P*>0.20, Student’s t-test, data not shown). In contrast, electroporation of Ct-SNAP-25 significantly and persistently reduced the magnitude of LTD (n = 11; 75±10% fEPSP slope of pre-stimulus baseline, *P*<0.05, Student’s t-test compared to control and scrambled peptide LTD). These data suggest that presynaptic Gβγ does play an essential role in the induction and expression of stimulus evoked LTD.

### Presynaptic infusion of the membrane-permeant Gβγ binding peptide mSIRK also blocks induction of stimulus-evoked LTD

To test by an independent method the hypothesis that Gβγ is a molecule necessary for the induction of presynaptic LTD, we utilized a different peptide known to bind selectively with high affinity to Gβγ known as mSIRK (**myr-SIRKALNIAGYPDYD-OH**; 47,48). Since mSIRK is cell-permeable, we extracellularly pressure ejected mSIRK (100 µM in ACSF plus 1% DMSO) at multiple sites covering *stratum pyramidale* throughout field CA3, and allowed 1-3 hour pre-incubation for mSIRK to diffuse into presynaptic Schaffer collateral terminals in field CA1, before attempting to induced Schaffer collateral-CA1 LTP. As shown in figure 6D, presynaptic infusion of mSIRK did not alter short-term depression immediately following LFS (2 Hz/10 min), but completely blocked induction of LTD (n = 6; 95±6% fEPSP slope of pre-stimulus baseline 60 min post-LFS) compared to slices where vehicle (ACSF plus 1% DMSO) alone was applied in field CA3 *stratum pyramidale* (n = 8; 75±3% fEPSP slope of pre-stimulus baseline; *P*<0.05, Student’s t-test), confirming the hypothesis that the interterminal release of Gβγ and the C-terminus of SNAP-25 are necessary for the induction of LTD.

### Presynaptic LTD is not due to persistent inhibition of presynaptic Ca^2+^ influx

P/Q and N-type calcium channels are the major source of action potential mediated Ca^2+^ influx into presynaptic boutons. Therefore, modulating the activity of these channels either intrinsically or by experimental manipulations could have direct effects on the release probability of synaptic vesicles. There is strong evidence to suggest that high voltage-activated calcium channels are downstream targets for presynaptic Gβγ proteins and the binding of these G-proteins leads to a voltage-dependent inhibition of calcium currents [49], [50]. Recent work has demonstrated that LTP of hippocampal perforant path-CA1 synapses can lead to an increase in release efficiency by enhanced recruitment of N-type calcium channels [51]. Thus, it is possible that LTD may employ a converse process of inhibiting VGCCs by binding of Gβγ that reduces Ca^2+^ influx and, hence, release probability. Thus, the reduction in LTD caused by scavenging of Gβγ by Ct-SNAP-25 (Fig. 6C) could be due to its lack of interaction with the C-terminus of SNAP-25, or with VGCCs, or both.

**Figure 7:**
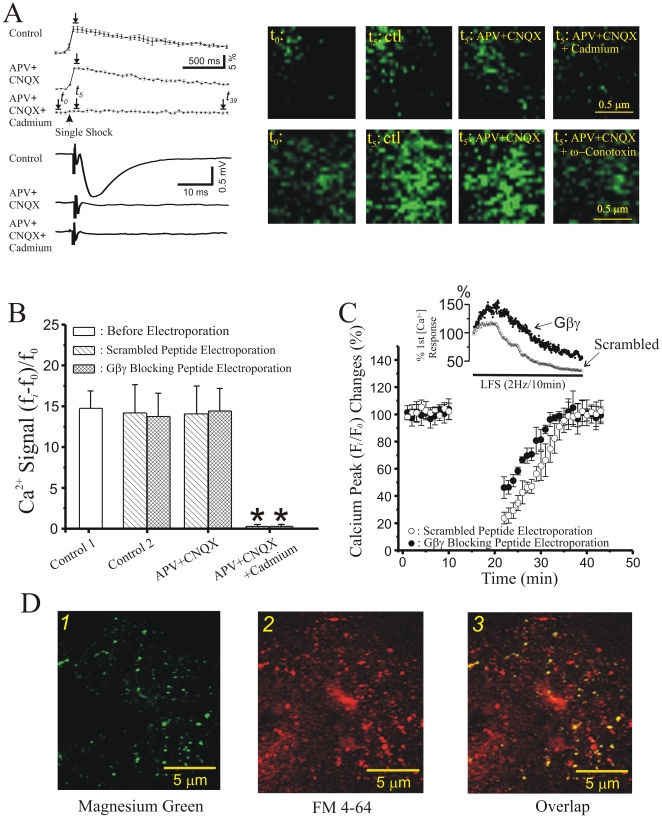
Presynaptic LTD at Schaffer collateral-CA1 synapses is not due to persistent inhibition of presynaptic calcium influx. **A**: Mean presynaptic stimulus-evoked Mg^2+^Green fluorescence changes in response to single electrical stimuli (top traces) and associated Schaffer collateral-evoked fEPSPs (lower traces) recorded in stratum radiatum of field CA1 after Mg^2+^Green-AM filling of presynaptic terminals. Images at the right are representative samples acquired using two-photon laser scanning microscope (top row) (bottom row). Single shock-induced increases in Mg^2+^Green fluorescence were not altered by either the NMDA receptor antagonist AP5 or the AMPA receptor blocker CNQX, but were completely blocked by adding Cd^2+^, indicating the signals were due to presynaptic Ca^2+^ influx through VDCCs. **B**: Mean ± SEM of fluorescence increases in Mg^2+^Green-AM filled presynaptic terminals evoked by individual Schaffer collateral stimuli before electroporation (open bar), versus 120 min after electroporating the CA3 pyramidal cell layer with either Ct-SNAP-25 (cross-hatched bar) or a scrambled control peptide (hatched bar). Cd^2+^ completely blocked these fluorescence signals, confirming that they were mediated by voltage-dependent Ca^2+^ channels. **C**: Time course of the effect of low frequency Schaffer collateral stimulation (LFS, 2 Hz/10 min, inset) on Schaffer collateral-evoked Mg^2+^Green fluorescence increases as % of pre-LFS responses, before, during, and after LFS, in terminals filled with Ct-SNAP-25 (filled circles; *n* = 4), versus terminals filled with scrambled control peptide (open circles; *n* = 4). **D**: Overlay of Mg^2+^Green stimulus-evoked [Ca^2+^] fluorescence increases with FM4-64 positive stimulus-loaded presynaptic terminals in the same field, co-localization that confirms their presynaptic nature.

To directly test whether presynaptic LTD is accompanied by a persistent inhibition of Ca^2+^ influx, we ejected Mg^2+^ Green-AM, a calcium indicator dye that is membrane-permeable [52], directly into the stratum radiatum of field CA1 of hippocampal slices. Mg^2+^ Green positive fluorescent puncta were visualized in field CA1 using two-photon excitation (Fig. 7D). Figure 7A demonstrates the kinetics of Mg^2+^ Green fluorescence increases in response to a single Schaffer collateral stimulus. These responses persisted in the presence of NMDA and AMPA receptor antagonists, despite the loss of fEPSPs, but were blocked by cadmium and omega conotoxin (Fig. 7A and B), consistent with a presynaptic nature for these calcium transients. To further confirm the presynaptic nature of these transients, FM4-64 was loaded into presynaptic terminals using 40 mM [K^+^]_o_ at the end of each experiment. Only those Mg^2+^ Green puncta that were also positive for FM4-64 fluorescence were analyzed by posthoc analysis (Fig. 7D).

To test whether there was a persistent inhibition of Ca^2+^ influx mediated by Gβγ proteins that might have contributed to LTD of transmitter release, we extracellularly injected Mg^2+^ Green-AM before electroporation of Ct-SNAP-25 or a scrambled control peptide. Electroporation itself did not alter presynaptic calcium signals, as the changes in normalized fluorescence intensity before and after electroporation were not significantly different (Fig. 7B, control; *P*<0.20, Student’s t-test compared to untreated controls). Following electroporation of either Ct-SNAP-25 or scrambled peptide, basal calcium signals in Schaffer collateral terminals were also not altered (data not shown). Next, we monitored the effect of inducing LTD on stimulus-evoked presynaptic calcium transients. A 2 Hz/10minute LFS in Ct-SNAP-25 treated slices elicited a significantly larger increase in presynaptic Ca^2+^ influx early in the LFS, which decayed to approximately 50% by the end of the LFS (Fig. 7C, inset). Post-LFS, single shock evoked Ca^2+^ transients reversed to basal levels within 20 minutes in both Ct-SNAP-25 and scambled peptide treated slices (Fig. 7C). Finally, we examined the magnitude of Schaffer collateral-induced presynaptic Ca^2+^ transients before and after bath application of BoNT/A (200 ng/ml), and found that presynaptic Ca^2+^ influx was not significantly altered by cleavage of the C-terminus of SNAP-25 (Fig. 7B, Pre BoNT/A peak Δ[Ca^2+^]  = 1.21±0.10, Post BoNT/A peak Δ[Ca^2+^]  = 1.24±0.13; *P*>0.20, paired t-test).

Taken together, these data indicate that, while endogenous Gβγ probably plays a role in the transient inhibition of voltage-gated calcium channels during and shortly after application of LFS, this interaction is reversible, does not depend on the C-terminus of SNAP-25 and cannot account for the persistent decrease in vesicular release associated with the expression of LTD.

## Discussion

In the hippocampus, inhibition of vesicle exocytosis by presynaptic GPCR activation is believed to be mediated principally via Giα subunits that inhibit adenylate cyclase in presynaptic terminals. We have previously demonstrated that, in acute hippocampal slice preparations, cAMP and cGMP exercise antagonistic roles in long-term synaptic plasticity at presynaptic loci [25], [26]. Stimulation of either class II metabotropic glutamate receptors (mGluRs) or A1 adenosine receptors, both of which are negatively coupled to adenylate cyclase, led to a transient synaptic depression, but when coupled with a simultaneous increase in [cGMP], elicited robust presynaptic long-term synaptic depression that persisted for more than 2 hours [25]. In another study using transgenic mice that express a constitutively active inhibitory G protein, Gαi2, we found that constitutive inhibition of adenylate cyclase enhanced the magnitude of stimulus-evoked LTD at Schaffer collateral-CA1 synapses [53].

However, it was unknown whether the Gβγ subunits that are concomitantly released upon the activation of these inhibitory GPCR’s [28] play any role in inducing long-term presynaptic plasticity. Recent work by Alford and colleagues [29], [30], [45] in the lamprey giant reticulospinal synapse has shown that serotonin-mediated presynaptic inhibition of neurotransmision is produced via Gβγ subunits acting downstream of Ca^2+^ entry [45]. Studies in the lamprey and mammals suggests that the C-terminus of SNAP-25, a region of the SNARE protein also know to interact with synaptotagmin 1 [54], is the target binding site for Gβγ that causes serotonin mediated presynaptic inhibition of transmitter release [29] At the same time, Gβγ subunits are also known to directly bind to alpha subunits of voltage-gated calcium channels and alter channel properties to decrease Ca^2+^ influx into the presynaptic terminal, an alternative mechanism that could also be responsible for reducing transmitter release probability [45], [49], [55], [56]. However, in the lamprey it has been shown that neither serotonin nor direct injection of Gβγ into the terminal produces a significant change in presynaptic Ca^2+^ influx, suggesting that Gβγ mediated inhibition is downstream of Ca^2+^ entry [57].

In the present study, we investigated whether Gβγ subunits play a necessary role in the expression of LTD at Schaffer collateral-CA1 synapses in the mammalian hippocampus, and whether the C-terminus of SNAP-25 might be a necessary binding target for Gβγ. Treatment of hippocampal slices with BoNT/A (200 ng/ml), a protease that selectively cleaves the 9 amino acid C-terminus of SNAP-25 [58], led to an ∼50% reduction in basal synaptic transmission which mean-variance analysis showed is due to decreasing initial vesicle release probability, probably by uncoupling synaptotagmin 1 binding to the SNARE complex [59]. This is consistent with other reports showing that BoNT/A cleavage of SNAP-25 does not completely abolish exocytosis [32], [59].

We found that, in addition to reducing basal release, BoNT/A also produced a selective and complete inhibition in the induction of LTD by low frequency (2 Hz) Schaffer collateral stimulation, while *de novo* LTP induced by theta burst stimulation was surprisingly unaffected by BoNT/A. Furthermore, saturation of LTD by repeated low frequency trains occluded the ability of BoNT/A to reduce synaptic transmission, strongly suggested that presynaptic LTD and BoNT/A converge on a common mechanism. Our data indicate that the C-terminus of SNAP-25 is critical for converting short-term synaptic depression into long-term synaptic depression, but is not required for induction of LTP, at least by the theta burst paradigm, implying that LTD is not always a reversal of mechanisms expressing LTP. To our knowledge, this is the first evidence of an essential role for the C-terminus of SNAP-25 in the expression of LTD of synaptic transmission.

This brings up another intriguing question; can BoNT/A treated synapses undergo bidirectional plasticity? We found that induction of depotentiation with a LFS given 15 min after inducing LTP could reverse LTP to almost pre-BoNT/A treated baseline levels, indicating that LTD and depotentiation use different expression mechanisms, with only LTD depending on the C-terminus of SNAP-25. In contrast, induction of LTP to reverse saturated LTD restored sensitivity of synaptic transmission to BoNT/A, indicating that, while LTP does not require the presence of the C-terminus of SNAP-25, it can restore fully depressed transmission to a place where it is again sensitive to cleavage of this portion of the molecule.

It was possible that BoNT/A impaired the expression of LTD simply by reducing basal release probability to a floor level. To investigate this possibility, we directly elevated extracellular [Ca^2+^] and tested the expression of LTD in both control and BoNT/A treated slices, with the magnitude of the increase in [Ca^2+^]_o_ selected to return evoked responses to their pre-BoNT/A amplitudes. While control slices were able to exhibit robust LTD under these conditions, BoNT/A treated slices still showed an ∼65% reduction in LTD amplitude, indicating that it was not simply the reduction in release probability, but the absence of the C-terminus of SNAP-25 as a molecular target, that was responsible for observed blockade of LTD. In fact, the continued expression of LTP, ability of depotentiation to reverse recent LTP, and of elevations in [cGMP] concentration to reduce presynaptic release, all indicate that Ct-SNAP-25 is a selective target in the expression of *de novo* LTD of vesicular release at Schaffer collateral-CA1 synapses.

Gβγ subunits are known to bind the C-terminus of SNAP-25 at a site used by synaptotagmin 1 [29], [57]. At modest calcium concentrations, the binding of Gβγ is believed to compete with synaptotagmin 1 for binding to SNAP-25, inhibiting vesicle exocytosis [54], and this mechanism underlies the reversible presynaptic depression produced by serotonin at Lamprey synapses [54]. Our current findings that electroporation of CA3 pyramidal neurons and their presynaptic terminals with the 14 amino acid C-terminus sequence of SNAP-25 partially blocked the induction of LTD, supports the conclusion that soluble presynaptic binding partners of this sequence are necessary for the full expression of LTD. The fact that this Ct-SNAP-25 peptide also blocked the presynaptic effects of group II mGluR activation, receptors known to liberate Gβγ, suggests that it is Gβγ that interacts with SNAP-25. Finally, our data showing that presynaptic infusion of CA3 pyramidal neurons with the cell-permeant Gβγ scavenging peptide mSIRK also blocked the induction of LTD confirms that, while it is certainly possible that an as yet unidentified molecule that shares the binding profile of Gβγ could still be responsible, the most likely candidate SNAP-25 binding molecule necessary for LTD is Gβγ. However, since activating multiple GPCRs that liberate Giα and Gβγ are able to elicit only *transient* presynaptic depression [25], [28], but not LTD, there must be an additional messenger system activated that converts the Gβγ-mediated depression to LTD.

Gβγ could also be involved in the expression of LTD by decreasing Ca^2+^ influx through direct binding to voltage-gated calcium channels, thereby reducing vesicular release probability. However, using imaging of presynaptic Ca^2+^ influx with Mg^2+^ Green-AM, we found that induction of LTD was associated with only a transient decrease in stimulus-evoked Ca^2+^ influx that returned to baseline level 20 minutes post-LFS, at a time when LTD was still robustly expressed. While short-term plasticity of release is likely to involve Gβγ-mediated suppression of voltage-dependent calcium channels, presynaptic LTD is not a result of long-term decreases in presynaptic [Ca^2+]^_i_.

An intriguing observation is that strong LTP could still be induced after treatment with BoNT/A, suggesting that the C-terminus of SNAP-25 is not involved in the expression of this form of LTP. Furthermore, since BoNT/A completely blocked induction of LTD, while Ct-SNAP-25 infusion only reduced LTD by ∼50%, it could either be that an insufficient concentration of Ct-SNAP-25 was achieved presynaptically to completely scavenge Gβγ, or that the SNAP-25 C-terminus liberated by BoNT/A might directly bind to targets necessary for the full expression of LTD. While this sequence has been shown to interact with and inhibit P/Q-type or N-type calcium channels [60], [61], our Mg^2+^ Green measurements do not support a *long-term* reduction in Ca^2+^ influx, though other presynaptic binding targets remain possibilities. Another alternative is that the remaining LTD may be both induced and expressed postsynaptically. Indeed, recent studies have highlighted a possible role for postsynaptic SNAP-25 in NMDA receptor trafficking [62] and mediating internalization of kainate receptors which results in LTD of kainate receptor-mediated EPSCs at mossy fiber-CA3 synapses [63].

If Gβγ is directly responsible for inhibiting vesicular release in LTD by binding SNAP-25, then the receptors that contribute these G proteins are likely to be in close proximity to the SNARE complex. Previous work from our lab has shown that either group II mGluR or adenosine A1 receptor activation, both of which are negatively coupled to adenylate cyclase and liberate Giα and Gβγ, can transiently reduce transmitter release and also enhance the induction of LTD [25], [27]. In this study, activation of group II mGluR’s by DCG-IV produced a depression of fEPSP’s that was markedly reduced by filling presynaptic terminals with Ct-SNAP-25, consistent with previous studies showing that Gβγ released from these receptors mediates this short-term depression. However, the fact that all of these GPCRs that liberate Giα and Gβγ typically elicit transient depression of release indicates that there must be additional factor(s) supplied by low-frequency stimulation. Our previous work has supplied evidence that one of these signaling pathways is the postsynaptic generation of the intercellular messenger NO, which diffuses to presynaptic terminals and activates guanylyl cyclase to generate cGMP [21]. Activation of PKG then liberates calcium from intracellular ryanodine receptor-gated stores [39], which results in activation of calcium-calmodulin kinase II, which is also a presynaptic requirement for generation of LTD [64].

A key unanswered question is what additional molecular events must occur to convert the short-term depression produced by Gβγ binding to SNAP-25 into LTD. Possible events could range from persistent dephosphorylation of Ser187 in the N-terminus of SNAP-25, which is known to reduce release probability, all the way to an unknown endogenous enzyme, which mimics BoNT/A in cleaving the C-terminus entirely. It is interesting to note that Ser187 in the native conformation of SNAP-25 is in close proximity to the C-terminus binding region of Gβγ [65], suggesting that Gβγ binding might open access of phosphatases to this site and switch the molecule to a dephosphorylated, lower release probability state. Understanding the molecular events underlying presynaptic LTD of vesicular release will be a necessary step in evaluating the role of presynaptic long-term plasticity in the consolidation of persistent changes in synaptic strength underlying memory storage and reconnection during subsequent learning.
